# Supplementary material for: Discontinuation of BRAF/MEK-Directed Targeted Therapy after Complete Remission of Metastatic Melanoma—A Retrospective Multicenter ADOReg Study
Source: Cancers (Basel). 2021 May 12;13(10):2312. doi: 10.3390/cancers13102312 (PMC8151093; doi:10.3390/cancers13102312)
Supplement: Supplementary file 1 [file cancers-13-02312-s001.zip › cancers-1206992-supplementary.pdf]

Supplementary Materials

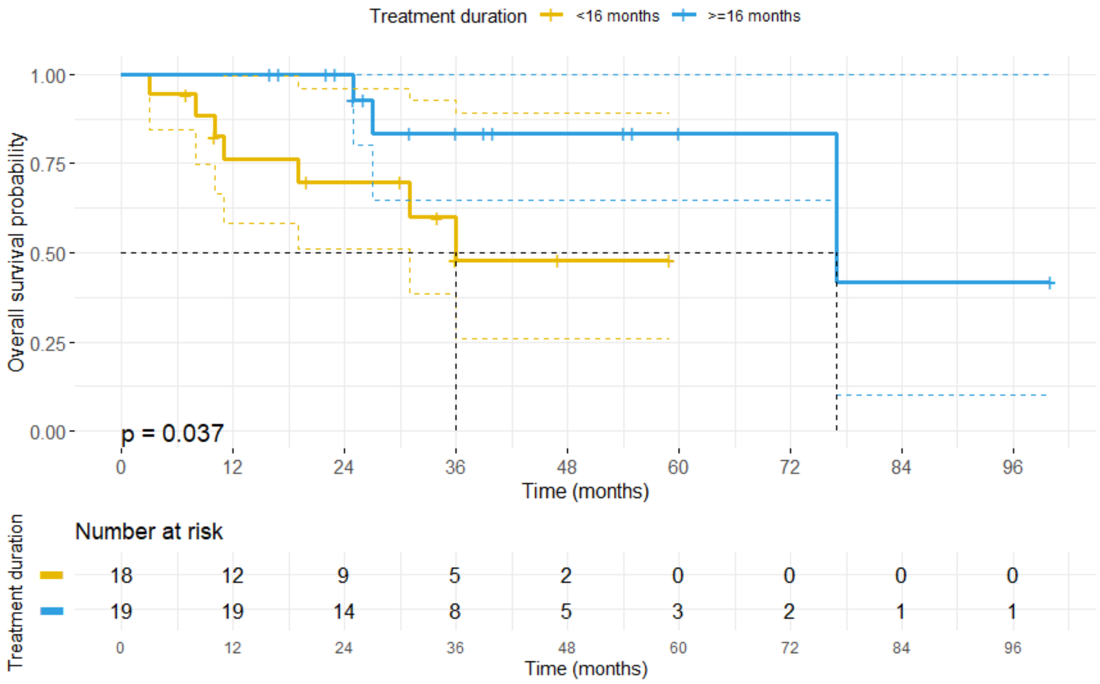

Figure S1: Initial duration of BRAF/MEKi-directed targeted therapy is correlated with the overall survival in complete responders. The Kaplan-Meier plot shows that patients with an initial duration of BRAF/MEKi therapy ≥16 months have a significantly longer overall survival (median OS: 77 months) as compared to patients with an initial TT treatment of less than 16 months (median OS: 36 months) (p=0,037).

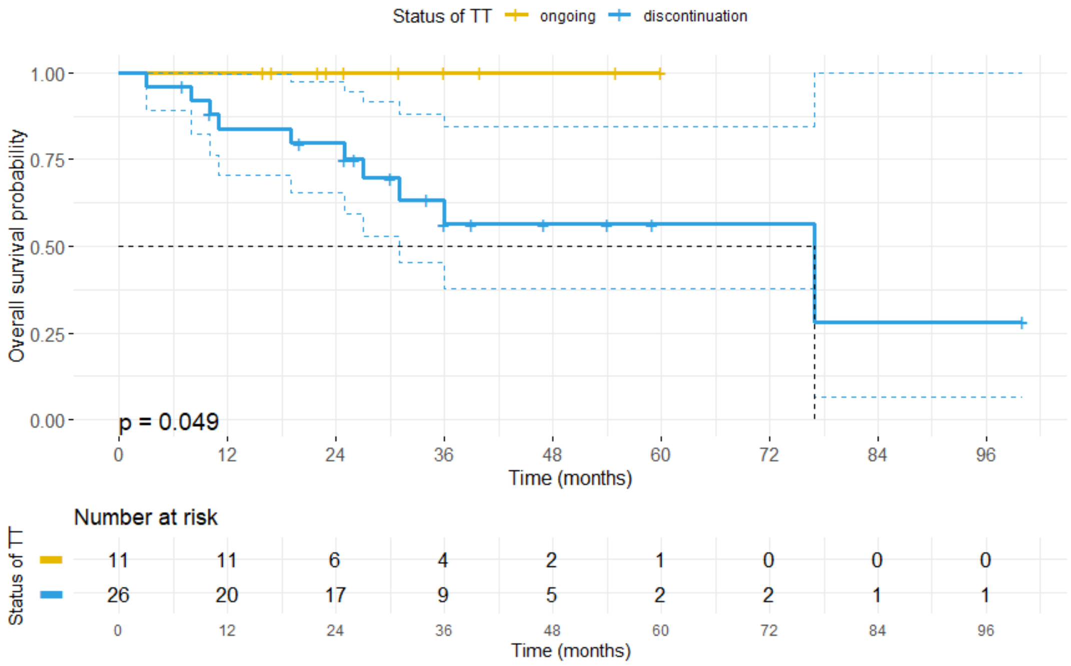

Figure S2: Patients with an initial CR ceasing BRAF/MEKi-directed targeted therapy have a shorter overall survival. Our data reveal that patients who have discontinued BRAF/MEKi therapy have a worse overall survival (median OS: 77 months vs. not reached) as compared to patients who have maintained BRAF/MEKi therapy throughout the entire observation period (p=0,049).

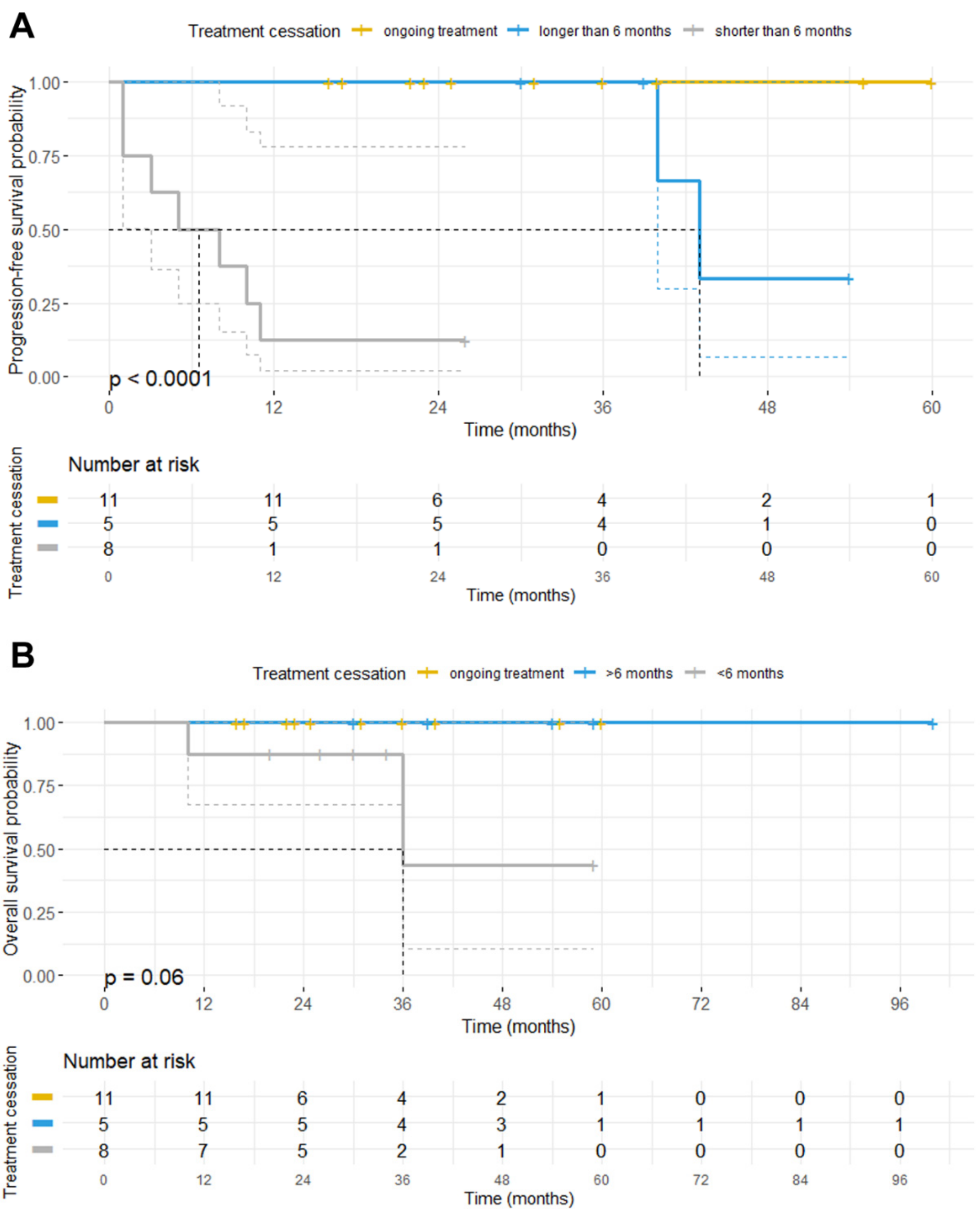

Figure S3: Patients maintaining their CR for a longer time interval after treatment cessation have a better progression-free and overall survival. Kaplan Meier plots depicting the progression-free (A) and overall survival (B) in patients ceasing BRAF/MEKi directed targeted therapy for other reasons than progressive disease. It can be found that patients who discontinued TT for a longer time interval than 6 months without showing tumor progression did have a significantly better PFS (33 months vs. 7 months) and OS (not reached vs. 36 months) as compared to patients who ceased BRAF/MEKi therapy for a shorter time interval, i.e., due to the exigency to re-initiate tumor treatment after tumor progression. Notably, patients ceasing TT for a longer time interval than 6 months did not have a shorter PFS, and OS as compared to patients receiving ongoing treatment.

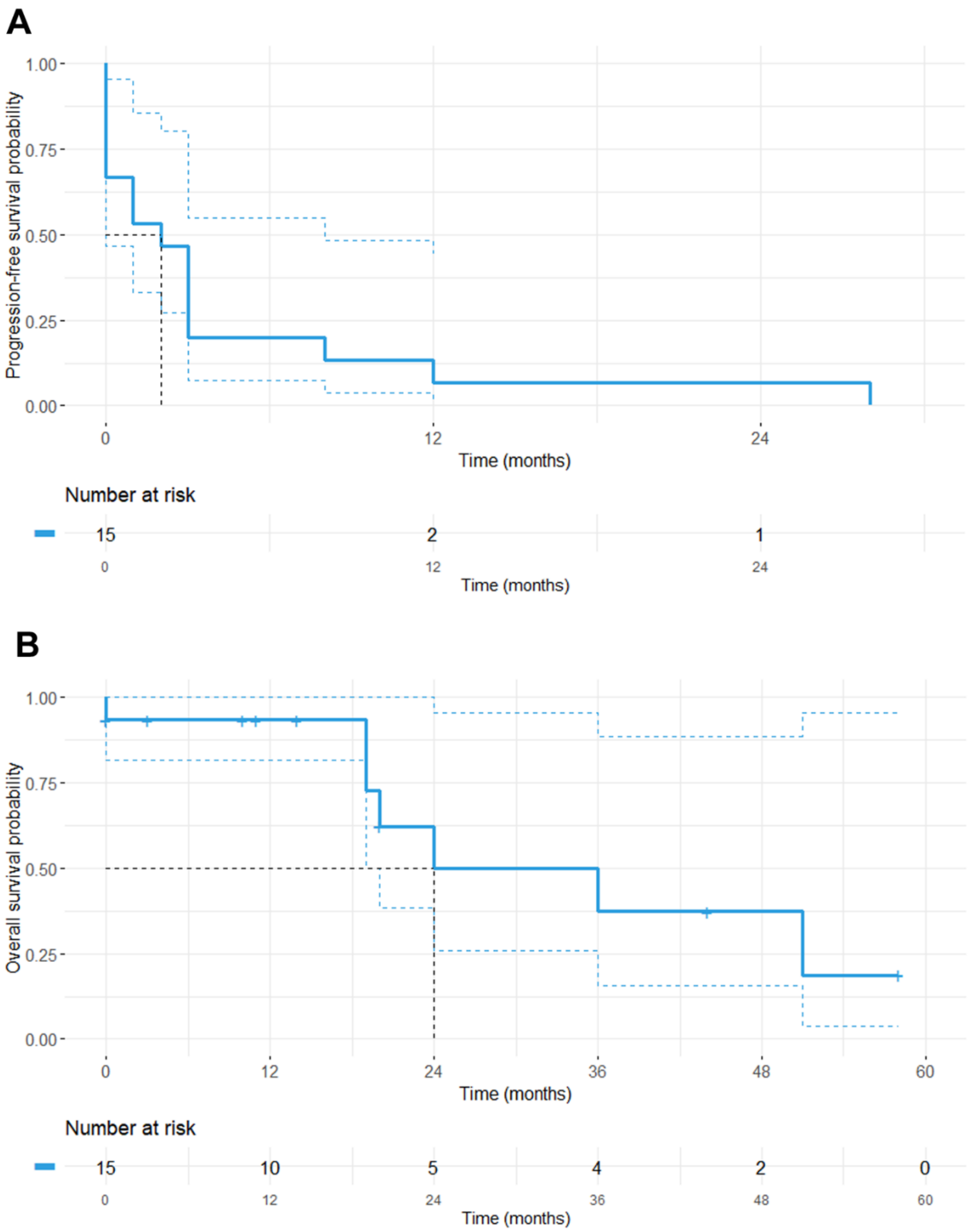

Figure S4. Progression-free and overall survival in patients receiving second line ICI treatment subsequent to tumor progression during first line BRAF/MEKi treatment. Patients receiving second line ICI-therapy after previous disease progression during BRAF± MEKi therapy showed a median PFS of 2 months (A) and a median OS of 24 months (B).
